# Decreased Ficolin-3-mediated Complement Lectin Pathway Activation and Alternative Pathway Amplification During Bacterial Infections in Patients With Type 2 Diabetes Mellitus

**DOI:** 10.3389/fimmu.2019.00509

**Published:** 2019-03-20

**Authors:** László József Barkai, Emese Sipter, Dorottya Csuka, Zoltán Prohászka, Katrine Pilely, Peter Garred, Nóra Hosszúfalusi

**Affiliations:** ^1^3rd Department of Internal Medicine, Semmelweis University, Budapest, Hungary; ^2^Laboratory of Molecular Medicine, Department of Clinical Immunology, Section 7631, Rigshospitalet, Faculty of Health and Medical Sciences, University of Copenhagen, Copenhagen, Denmark

**Keywords:** ficolin-3, lectin pathway, alternative pathway, classical pathway, complement, type 2 diabetes, bacterial infection, *Escherichia coli*

## Abstract

Bacterial infections are frequent and severe in patients with diabetes mellitus. Whether diabetes *per se* induces functional alterations in the complement system hampering activation during infection is unknown. We investigated key elements of the complement system during bacterial infections in patients with type 2 diabetes mellitus (T2DM) and compared them to non-diabetic (ND) individuals. Using a prospective design, we included 197 T2DM, and 196 ND subjects, all with clinical diagnosis of acute community-acquired bacterial infections. Functional activities of the ficolin-3-mediated lectin (F3-LP), mannose binding lectin-mediated lectin- (MBL-LP), classical (CP), and alternative pathways (AP), as well as concentrations of complement activation products C4d and sC5b-9 were determined. Functional *in vitro* activities of F3-LP and AP were significantly higher in T2DM than in ND subjects, (median 64% vs. 45%, *p* = 0.0354 and 75 vs. 28%, *p* = 0.0013, respectively), indicating a decreased *in vivo* activation and lack of consumption of F3-LP and AP in T2DM patients, whereas no difference in functional capacities of CP and MBL-LP were observed between T2DM and ND subjects. Diminished F3-LP and AP activation was most pronounced in diabetic patients with urinary tract infections with positive microbiological culture results for *Escherichia coli* bacteria. In the T2DM group 3-months mortality significantly associated with diminished F3-LP and AP, but not with CP activation. Concentrations of C4d and sC5b-9 were significantly lower in the T2DM than in ND patients. In conclusion, we found impaired F3-LP activation and lack of AP amplification during bacterial infections in patients with type 2 diabetes, compared to non-diabetic subjects, suggesting a diminished complement mediated protection to bacterial infections in T2DM.

## Introduction

Diabetes is considered as an independent risk factor for community acquired and nosocomial infections (1). It increases the susceptibility to infectious diseases, moreover, prolongs the infection-related hospitalization and may enhance its mortality (2, 3). The background of this acquired immunodeficiency is not completely clear (4, 5). Regarding innate immunity, *in vitro* studies identified polymorphonuclear neutrophil (PMN) dysfunction including impaired PMN transmigration through barriers (6), reduced PMN chemotaxis (7) and as the most convincing evidence, decreased microbial killing (7–9). Available data are controversial for the adaptive immunity. T-lymphocyte dysfunction seems to be dependent on glycemic control, as T cell proliferation was impaired in poorly controlled patients with type 1 diabetes (10). By contrast, patients with relatively good metabolic control showed a robust secondary immune response to standard antigens (11). With regard to humoral immunity, glycation may impair the biological function of antibodies (12).

Only a few data are known about the activation of the complement system in bacterial infections in diabetes. Nevertheless, complement activation has been shown to be a contributing factor to complications of diabetes (13). C3 as a central component of complement and its activation may contribute to diabetic nephropathy, retinopathy and neuropathy (13–17). Regarding the macrovascular complications, Hess and colleagues showed the possible role of C3 in diabetes related cardiovascular risk, by proposing a mechanism in which C3 participates in a hypofibrinolytic, and thus prothrombotic state (18). In a recent review, Ghosh and colleagues summarized the body of evidence supporting the role of the complement system and complement regulatory proteins in the pathogenesis of diabetic vascular complications, with specific emphasis on the role of the membrane attack complex (MAC) and of CD59, an extracellular cell membrane-anchored inhibitor of MAC formation that is inactivated by non-enzymatic glycation (19).

On the other hand a diminished complement-activating capacity through the classical pathway in type 2 diabetes mellitus was reported in the context of free sialic acid as a potential modulator of complement activation (20). Regarding the effect of high glucose on complement activation, *in vitro* assays showed that classical and alternative pathway activities were not affected by elevated glucose or other hexoses tested (21). However, high glucose concentrations inhibited the complement activation via the mannose binding lectin (MBL) mediated pathway (21). The role of the complement system in infectious diabetic complications has been studied scarcely.

Ficolins−1,−2,−3 and mannose binding lectin are pattern recognition molecules playing an important role in activating the lectin complement pathway (22–24). MBL binds directly to high mannose or fucose structures on microbial surfaces and drives activation of the lectin pathway (25). Ficolin-1 and ficolin-3 were shown to bind carbohydrate structures of bacteria, especially N-acetyl-galactosamine, and N-acetyl-D-glucosamine, additionally ficolin-3 can associate also with glucose and fucose (26). Ficolin-2 is the major 1,3-β-glucan-binding protein in human plasma and can bind to lipoteichoic acid, thus, ficolin-2 may bind to a wide variety of fungi and Gram-positive bacteria (27, 28). Two patients with congenital ficolin-3 deficiency were reported suffering from serious and life-threatening infections caused by *Haemophilus influenzae* and *Pseduomonas aeruginosa* (29), and necrotizing colitis (30). Despite these data, previously, ficolin-3 was identified to bind only a few common pathogenic bacteria (31). Moreover, its potential to activate the lectin pathway *in vivo*, and to augment phagocytosis, has been described only for the opportunistic bacteria, *Hafnia alvei* (32) until now. The role of ficolins in infections of patients with diabetes was not studied earlier, despite that MBL, and ficolins recognize specific carbohydrate patterns expressed by microorganisms, and elevated blood glucose and/or protein glycation seen in diabetes may alter such carbohydrate patterns.

Therefore, the aim of our study was to investigate complement activation and consumption via the alternative, classical and lectin pathways during bacterial infections in patients with type 2 diabetes (T2DM). Accordingly, the objective of our study was to provide complex observational data on the lectin pathway focusing on the ficolin-3-mediated pathway in diabetes mellitus in the context of community acquired bacterial infections.

## Materials and Methods

### Patients

In this prospective, observational study patients with clinical diagnosis of bacterial infections were enrolled and divided into two groups according to the presence [T2DM group, based on WHO criteria (33) or absence of type 2 diabetes mellitus (non-diabetic, ND group)]. Inclusion criteria were a minimum age of 18 years and the need for hospitalization on a general medicine ward due to acute community-acquired bacterial infection. Patients with hematological, oncological or immunological illnesses were excluded. Sepsis was defined in case of at least two existing SIRS (systemic inflammatory response syndrome) criteria from the following: 1. Temperature >38°C (100.4°F) or <36°C (96.8°F), 2. Heart rate >90/min, 3. Respiratory rate >20/min or PaCO2 <32 mm Hg, 4. WBC >12.000/mm^3^, <4.000/mm^3^, or >10% bands (34, 35). To ascertain post infection mortality rates, subjects were followed-up after a 3-months period through their social insurance identification number, and via interview on their-, or their family member's telephone number.

### Data Collection

Clinical data of current symptoms and past medical history were assembled from the patients' medical records and a further thorough interview after admission to the hospital.

### Blood Sampling

Samples (serum, EDTA-anticoagulated plasma and sodium-citrate-anticoagulated plasma) of both groups were collected from antecubital venipuncture within the first 3 days of the hospitalization, between September 2013 and December 2016. Cells and supernatants were separated by centrifugation at 2000x g and the aliquoted serum and plasma samples were stored at −70°C until analysis.

### Determination of Complement Parameters

For functional assessment of either the alternative pathway (AP, reference range based on the values of healthy blood donors: 70–125%), or the MBL-mediated lectin pathway activation (MBL-LP, measured by MBL activation with mannan, reference range: 30–130%), as well as the ficolin-3-mediated lectin pathway activation (F3-LP, measured by ficolin-3 activation with acetylated BSA, reference range: 25–130%) in serum samples, commercial ELISA kits (Wieslab, Eurodiagnostica, Malmö, Sweden) detecting generation of terminal complement complex (C9 neo-epitope) were used, according to the manufacturer's instructions. Total classical pathway activity (CP, reference range: CH50 48–103 U/mL) was assessed with a home-made sheep-erythrocyte hemolytic titration test, using serum samples. In these standardized assays, the decreased or absent *in vitro* functional activity of complement pathways may be related to *in vivo* consumption (36). The concentrations of ficolin-1 (F1, reference range: 10–1,890 ng/mL) (37), ficolin-2 (F2, reference range: 1.00–12.20 μg/mL) (38), ficolin-3 (F3, reference range, 3–54 μg/mL) (39) and MBL (reference range: 0–5,000 ng/mL) (40) were determined in serum by standard sandwich ELISA techniques: monoclonal antibodies specific for each protein were coated on the plates, and after the incubation of the samples, biotinylated antibodies were applied, and streptavidin/HRP complexes were used for detection.

Complement C3 (reference range: 0.90–1.80 g/L) and C4 (reference range: 0.15–0.55 g/L) levels were measured in serum by turbidimetry (Beckman Coulter, Brea, CA), whereas, radial immunodiffusion was performed to measure the antigenic concentration of C1-inhibitor (reference range: 0.15–0.30 g/L) using polyclonal goat anti-human C1-inhibitor (Quidel, San Diego, CA, USA). Commercial ELISA kits were used to quantify the levels of C4d (reference range: 0.70–6.30 μg/mL) and sC5b-9, the soluble form of the terminal pathway activation complex (reference range: 110–252 ng/mL); both complement activation products were measured in EDTA-plasma (Quidel, San Diego, CA, USA).

### Other Laboratory Measurements

C-reactive protein (hsCRP) levels were ascertained by turbidimetry (Beckman Coulter, Brea, CA), other clinical laboratory parameters were measured with Beckman Coulter (Brea, CA) or the Cell-Dyn 3,500 hematology analyzer. Blood glucose level was determined using the hexokinase assay. Fructosamine was measured with Roche Fructosamine colorimetric test kit (using nitrotetrazolium blue chloride on a Beckman Analyzer AU680, reference range: 205–280 μmol/L). HbA_1c_ concentration was quantified with ion exchange high pressure liquid chromatography (HPLC) method (reference range: 4.0–6.0%). Advanced glycation end product (AGE) levels of both groups were measured in skin by non-invasive autofluorescence technique (AGE Reader mu, DiagnOptics); according to the manufacturer's instructions (41).

### Statistical Analysis

Statistical calculations were carried out using GraphPad Prism 5 (Graphpad Software, USA; www.graphpad.com). Results are presented as medians with 25–75% percentile, or numbers (percentage). To compare variables in two independent groups Mann-Whitney test or chi-square test, to analyze associations between variables Spearman correlation was used. All statistical analyses were two-tailed, significance threshold was set at p = 0.05.

## Results

### Clinical Characteristics of the Two Cohorts

A total of 197 T2DM and 196 ND subjects were included; clinical characteristics of the two groups are presented in Table 1. Blood glucose, glycation parameters reflecting short-, middle-, and long-term blood glucose levels (fructosamine, HbA_1c_, AGEs) and BMI were significantly higher (all *p* < 0.001) in the T2DM group compared to the ND. No differences were found between the two groups regarding age and gender distribution. Infection-related laboratory markers (C-reactive protein, white blood cell count) were equally, substantially elevated in both groups (average CRP concentration well-above 100 mg/L, and WBC above 12 G/L), with at least half (54–60%) of subjects with sepsis (Table 1).

**Table 1:** Clinical characteristics of the T2DM and the ND group.

**Clinical characteristics**	***T2D*M (*n* = 197)**	***N*D (*n* = 196)**	**Significance**
**Females** (%)	**52**	**55**	NS
**3-month survival** (%)	**83**	**80**	NS
^*^Age (years)	**73** [64.0–80.0]	**73** [60.0–82.0]	NS
^*^Blood glucose on admission (mmol/L)	**10.7** [7.8–15.8]	**6.6** [5.8–7.8]	***p*** **<** **0.0001**
^*^Fructosamine (μmol/L)	**235.3** [208.7–314.6]	**192.4** [169.1–205.4]	***p*** **<** **0.0001**
^*^HbA_1c_ (%)	**7.5** [6.4–8.7]	**5.6** [5.3–6.0]	***p*** **<** **0.0001**
^*^AGEs- skin autofluorescence (AU)	**1.172** [1.1–1.4]	**1.065** [0.9–1.2]	***p*** **=** **0.0004**
^*^BMI (kg/m^2^)	**28.4** [25.1–33.5]	**26.0** [22.0–29.4]	***p*** **<** **0.0001**
^*^Diabetes duration (years)	**14.0** [6.0–23.8]	n/a	n/a
^*^CRP (mg/L)	**129.6** [61.3–228.8]	**142.5** [84.6–220.9]	NS
^*^WBC (G/L)	**12.5** [9.6–17.2]	**13.8** [10.4–18.2]	NS
**Prevalence of sepsis on admission** (%)	**54**	**60**	NS

*Mortality rate: within 3 months after infection, BMI, Body mass index; AGEs, Advanced Glycation End products; AU, Arbitrary Unit; CRP, C-reactive protein; WBC, white blood cell count (total)*.

Sepsis was defined in case of at least two existing SIRS (systemic inflammatory response syndrome) criteria from the followings:

*1. Temp >38°C (100.4°F) or <36°C (96.8°F), 2. Heart rate > 90/min, 3. Respiratory rate >20/min or PaCO2 <32 mm Hg, 4. WBC > 12,000/mm^3^, <4,000/mm^3^, or > 10% bands*.

* *Values indicate medians [25–75% percentile] of the variables*.

*For comparison of the two groups Mann-Whitney test or chi-square test was performed*.

*The values in bold indicate the medians and the significant differences*.

### Complement Parameters in the Study Groups

In order to get a reasonably detailed profile on the complement system, concentrations of recognition molecules (ficolin-1,−2,−3, and MBL), levels of C1-inhibitor, C3, C4, C4d, sC5b-9, as well as functional activity of the ficolin-3- or MBL-mediated lectin pathway activation (referred to as F3-LP, or MBL-LP, respectively), and of the classical (CP) and alternative (AP) pathways were determined. As shown in Table 2 patients with bacterial infections but without diabetes (ND) had marked, strong complement activation, as reflected by the very high terminal pathway activation marker sC5b-9 levels. Patients with type 2 diabetes (T2DM) during infection had also elevated but significantly lower sC5b-9 concentrations when compared to ND (*p* = 0.0022). Regarding the complement activity of various complement pathways, diabetic patients and the ND group had similar classical pathway consumption (Table 2). In contrast, *in vitro* activation of F3-LP and AP were more pronounced in samples of patients with bacterial infections and diabetes in comparison with ND group, as reflected by the difference of F3-LP and AP activity (in average by 19–47% higher values observed by the *in vitro* activation test in T2DM, *p* = 0.0354, *p* = 0.0013, respectively) (Table 2). Note, that the higher *in vitro* complement pathway activation reflects a lower *in vivo* activation and/or consumption. The diminished *in vivo* activation of F3-LP and AP in diabetic patients with bacterial infections was not due to lower levels of ficolins or C4 as antigenic concentrations of these recognition molecules and complement components were similar in the two groups (Table 2). Concentrations of C3 were only slightly elevated in the T2DM group (*p* = 0.0482) (Table 2). No difference concerning MBL concentration and functional capacity of MBL-LP was observed between the two groups (Table 2). Importantly, diminished activation of F3-LP in patients with bacterial infections and diabetes was also supported by the significantly lower C4d concentrations, when compared to ND (*p* = 0.0063). Similarly, the significantly higher concentration of C1-inhibitor in the T2DM group compared to ND (*p* < 0.0001) may reflect the impaired activation and consumption of F3-LP (Table 2).

**Table 2:** Complement results of the T2DM and ND groups.

**Complement parameters (reference range)**	**T2DM *n* = 197**	**ND *n* = 196**	**Significance**
**Ficolin-3-mediated lectin pathway (F3-LP)** (—% 25–130%)	**64** [25-111]	**45** [16–92]	***p*** **=** **0.0354**
**F3 conc**. - μg/mL (3–54 μg/mL)	**36** [24–56]	**34** [22–52]	NS
**F1 conc**. - ng/mL (10–1890 ng/mL)	**558** [354–983]	**568** [327–943]	NS
**F2 conc**. - μg/mL (1.00–12.20 μg/mL)	**1.60** [1.01–2.39]	**1.50** [0.94–2.40]	NS
**MBL-mediated lectin pathway (MBL-LP)**–% (30–130%)	**73** [5–116]	**50** [5–111]	NS
**MBL conc**. - ng/mL (0–5000 ng/mL)	**982** [178–2082]	**888** [285–1963]	NS
**Alternative pathway (AP)** - % (70–125%)	**75** [18–98]	**28** [0–92]	***p*** **=** **0.0013**
**Classical pathway (CP)**–**CH50** - U/mL (48–103 U/mL)	**30.00** [13–63]	**30.00** [13–60]	NS
**C1-inhibitor** – g/L (0.15–0.30 g/L)	**0.30** [0.26–0.34]	**0.27** [0.22–0.31]	***p*** **<** **0.0001**
**C3 conc**. - g/L (0.90–1.80 g/L)	**1.87** [1.59–2.18]	**1.76** [1.46–2.17]	***p*** **=** **0.0482**
**C4 conc**. - g/L (0.15–0.55 g/L)	**0.43** [0.34–0.52]	**0.42** [0.33–0.53]	NS
**C4d conc**. - μg/mL (0.70–6.30 μg/mL)	**6.51** [4.27–10.75]	**7.87** [5.5–11.61]	***p*** **=** **0.0063**
**sC5b-9 conc**. - ng/mL (110–252 ng/mL)	**457** [341–643]	**516** [410–669]	***p*** **=** **0.0022**

*Values indicate medians [25–75% percentile] of the variables, conc.: concentration. Mann-Whitney test was performed to compare study groups*.

*The values in bold indicate the medians and the significant differences*.

**Figure 1:**
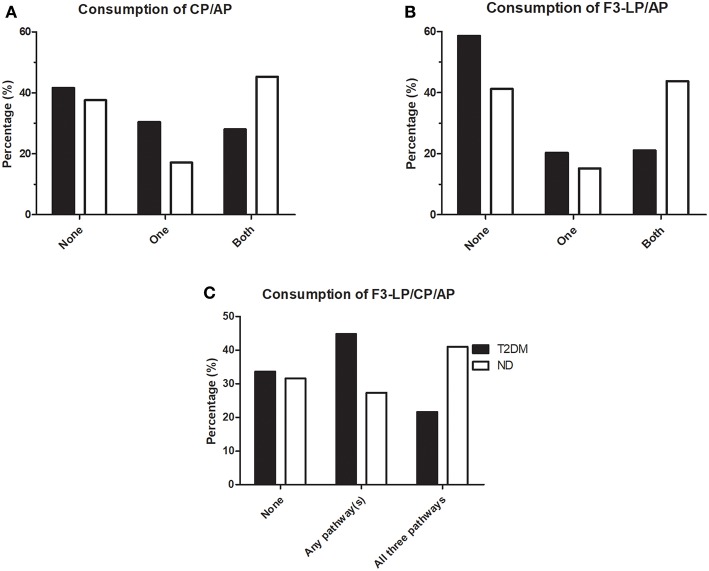
Occurrence of complement consumption in patients with bacterial infections, with (T2DM) or without (ND) diabetes mellitus. **(A)** Bars indicate percentages of patients without complement consumption (None), with consumption of only one or both pathways for CP/AP. (*p* = 0.0518, chi-square test). **(B)** Bars indicate percentages of patients without complement consumption (None), with consumption of only one or both pathways for F3-LP/AP. (*p* = 0.0007, chi-square test). **(C)** Bars indicate percentages of patients without complement consumption (None), with consumption of any of the three pathways indicated above the graph. (*p* = 0.037, chi-square test). Complement consumption was defined according to the lower limit of reference ranges, as indicated in Table 2. Chi-square test was performed, stratified according to T2DM/ND and occurrance of 0, 1, or more consumed complement parameters. (CP, classical pathway; AP, alternative pathway; F3-LP, ficolin-3-mediated lectin pathway).

To further explore the relationship between consumption of different pathways during bacterial infections in diabetic patients and non-diabetic individuals, analyses of parallel consumptions were done. Consumption of various complement pathways such as CP, AP and F3-LP were defined according to the lower limit of the reference range, as indicated in Table 2 (<48 U/mL, <70%, <25%, respectively). “No consumption” was considered if the values were equal or above the lower limit of the reference. As shown on Figure 1, distribution of complement consumption was polarized in non-diabetic patients: a significant percentage of ND patients fell into subgroups without any consumption (38% for CP and AP, 41% for F3-LP and AP) however, another significant percentage showed a consumption of both pathways (45% for CP and AP, 44% for F3-LP and AP) (Figures 1A,B). In contrast, diabetic patients with bacterial infections showed different patterns: 42% fell into subgroups without any consumption for CP and AP and 59% for F3-LP and AP; while only 28% had a consumption of both pathways for CP and AP and 21% for F3-LP and AP. The T2DM group had less frequent parallel consumptions of F3-LP and AP in comparison with the ND group (*p* = 0.0007) (Figure 1B), and a similar finding was observed for CP and AP (*p* = 0.0079) (Figure 1A). In addition, when analyzing all three pathways together, 48% of ND patients, but only 27% of diabetic patients had parallel activation and consumption of all three pathways F3-LP, CP and AP (*p* = 0.037) (Figure 1C). This difference is mainly attributable to the diminished activation of F3-LP and AP in diabetes (Figure 1).

### Activation and Consumption of F3-LP and AP Among Different Types of Infections

To obtain a more detailed view on the diminished F3-LP and AP activation in diabetic patients with bacterial infections, subjects were allocated into subgroups, according to the anatomic location of infections: respiratory tract (Resp.), urinary tract (UTI), skin and soft tissue (SSTI), and infections in other locations.

The diminished *in vivo* activation (lack of consumption) of F3-LP and AP among diabetic patients was most pronounced in case of UTI (Tables 3, 4). Non-diabetic subjects had in average 30% lower *in vitro* functional activity of F3-LP than patients with diabetes (37% [8–79] vs. 67% [21–102], *p* = 0.0456). Slight difference alike was observed in patients with sepsis and UTI (27% [6–73] vs. 67% [20–102], *p* = 0.038, Table 3). Note, that the observed lower *in vitro* complement pathway activity reflects a higher *in vivo* activation and/or consumption, and the higher *in vitro* complement pathway activation means a lower *in vivo* activation and/or consumption, as previously described. Similar results were seen in the subgroups with respiratory infection, though the difference between the groups did not reach statistical significance (Table 3). Regarding SSTIs, SSTIs with sepsis, and other localization of infections, no significant differences in F3-LP activity were seen between the groups (Table 3). Similarly, to the F3-LP, *in vivo* activation of AP was diminished among diabetic patients having respiratory or urinary tract infections (*p* = 0.0276 and *p* = 0.0092, respectively, Table 4). These two locations represented the majority of patients in both study groups.

**Table 3:** Activity of ficolin-3-mediated lectin pathway (F3-LP) in relation to anatomic location of bacterial infections.

**Infection type**	**T2DM** ***n*** **=** **197**		**ND** ***n*** **=** **196**		**Significance**
**Respiratory**	**Total:** ***n*** **=** **46**	**67** [23–132]	**Total:** ***n*** **=** **74**	**44** [15–89]	NS
	**Sepsis:** ***n*** **=** **31**	**52** [12–103]	**Sepsis:** ***n*** **=** **49**	**39** [9–74]	NS
**UTI**	**Total:** ***n*** **=** **60**	**67** [20–102]	**Total:** ***n*** **=** **61**	**37** [8–79]	***p*** **=** **0.0456**
	**Sepsis:** ***n*** **=** **40**	**67** [20–102]	**Sepsis:** ***n*** **=** **43)**	**27** [6–73]	***p*** **=** **0.038**
**SSTI**	**Total:** ***n*** **=** **75**	**53** [25–100]	**Total:** ***n*** **=** **47**	**68** [18–114]	NS
	**Sepsis:** ***n*** **=** **31**	**53** [30–85]	**Sepsis:** ***n*** **=** **21**	**72** [10–123]	NS
**Other**	**Total:** ***n*** **=** **16**	**59** [21–136]	**Total:** ***n*** **=** **14**	**63** [21–117]	NS

*UTI, Urinary tract; SSTI, Skin and soft tissue infections. Due to low case number, sepsis subgroups for “Other” infections are not presented*.

*“n” indicates number of subjects. Values indicate medians [25–75% percentile] of F3-LP (%). Mann-Whitney test was performed to compare study groups*.

*The values in bold indicate the medians and the significant differences*.

**Table 4:** Activity of alternative pathway (AP) in relation to anatomic location of bacterial infections.

**Infection type**	**T2DM** ***n*** **=** **197**		**ND** ***n*** **=** **196**		**Significance**
**Respiratory**	**Total:** ***n*** **=** **46**	**77** [1–103]	**Total:** ***n*** **=** **74**	**17** [0–71]	***p*** **=** **0.0276**
	**Sepsis:** ***n*** **=** **31**	**68** [0–101]	**Sepsis:** ***n*** **=** **49**	**6** [0–72]	NS
**UTI**	**Total:** ***n*** **=** **60**	**73** [18–98]	**Total:** ***n*** **=** **61**	**22** [0–83]	***p*** **=** **0.0092**
	**Sepsis:** ***n*** **=** **40**	**67** [8–96]	**Sepsis:** ***n*** **=** **43**	**17** [0–91]	NS
**SSTI**	**Total:** ***n*** **=** **75**	**77** [18–89]	**Total:** ***n*** **=** **47**	**82** [0–102]	NS
	**Sepsis:** ***n*** **=** **31**	**48** [6–81]	**Sepsis:** ***n*** **=** **21**	**6** [0–102]	NS
**Other**	**Total:** ***n*** **=** **16**	**68** [27–110]	**Total:** ***n*** **=** **14**	**32** [0–106]	NS

*UTI, Urinary tract; SSTI, Skin and soft tissue infections. Due to low case number, sepsis subgroups for “Other” infections are not presented*.

*“n” indicates number of subjects. Values indicate medians [25–75% percentile] of AP (%). Mann-Whitney test was performed to compare study groups*.

*The values in bold indicate the medians and the significant differences*.

**Figure 2:**
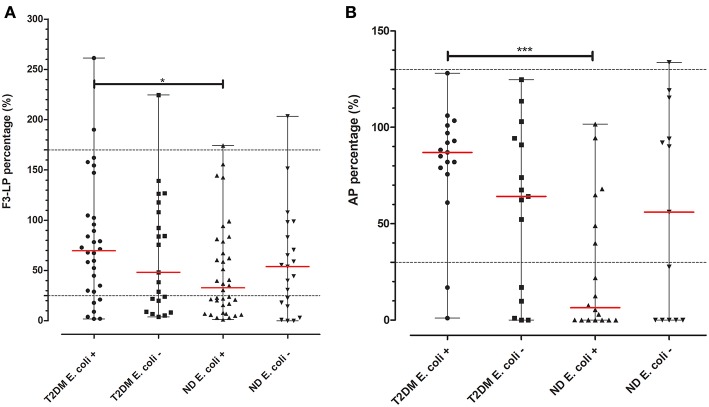
F3-LP and AP activity in subjects with UTI and a positive microbiological culture. Ficolin-3-mediated lectin **(A)** and alternative **(B)** pathway activations (*in vitro*) of patients with UTIs and positive urine and/or blood cultures were chosen from both cohorts. Depending on the presence or absence of the *E. coli* bacterium they were each once more divided into two subgroups. T2DM subjects with *E. coli* positive cultures had higher *in vitro* F3-LP levels than those of the ND cohort (70% [30–103] vs. 33% [16–91], *p* = 0.0286). Similar results were found regarding AP (87% [77–99] vs. 6% [0–53], *p* = 0.0003). Difference between the groups was analyzed with the Mann-Whitney test, *p < 0.05, and ***p < 0.001. Horizontal dashed lines indicate limits of reference ranges, horizontal red lines indicate median values of the variables.

Based on the pronounced difference in F3-LP and AP activity, we aimed to further analyze the subgroup with UTI. As shown on Figure 2, diabetic patients with positive culture results for *Escherichia coli (E. coli)* had diminished F3-LP activation when compared to those of non-diabetic subjects (70% [30–103] vs. 33% [16–91], *p* = 0.0286). Similar results were observed regarding AP (87% [77–99] vs. 6% [0–53], *p* = 0.0003). However, no difference concerning F3-LP and AP was observed for those, with non-*E. coli* UTIs. (Figure 2).

### Association of Complement Activation and Consumption With Clinical Parameters

To determine the relationship of complement activation and consumption with clinical parameters including glycation-related markers in T2DM, Spearman correlation analysis was done. No associations of F3-LP or AP with blood glucose, fructosamine, or HbA_1c_ were found, however, an inverse weak correlation of F3-LP with the long-term glycation marker of AGEs (*p* < 0.05, *r* = −0.2765) was observed among T2DM subjects.

**Figure 3:**
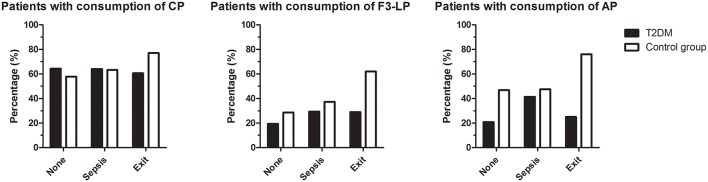
Occurrence of complement consumption in patients with bacterial infections, with (T2DM) or without (ND) diabetes mellitus. Bars indicate percentages of patients with consumption of classical (left), ficolin-3-mediated lectin (middle), or alternative (right) pathways, according to subgroups with or without sepsis and mortality within 3 months. The group 'exit' includes all patients who died, irrespective of the presence of sepsis. Left panel: *p* = 0.278, middle: *p* = 0.012, right: *p* = 0.025, chi-square test.

Three-months mortality after bacterial infection was similar in the diabetic and non-diabetic groups (Table 1), however, lack of F3-LP activation and consumption with lack of AP amplification were associated with mortality in the diabetic group (*p* = 0.012 and *p* = 0.025, respectively) (Figure 3). Whereas, activation of CP, F3-LP and AP was present in 77, 62, and 76% of ND subjects who died, respectively, the same was not observed for T2DM patients. Although 60% of those diabetic patients who died had CP activation and consumption, this proportion was only 29 and 25% for F3-LP and AP, respectively (Figure 3). There was no difference in the occurrence of complement consumption among the groups with sepsis only (Figure 3).

## Discussion

In our prospective study, we found decreased *in vivo* activation of ficolin-3-mediated lectin and alternative pathways during bacterial infections in patients with type 2 diabetes in comparison with non-diabetic subjects. On the other hand, there was no difference between the two study groups regarding the classical pathway and the MBL-mediated lectin pathway activities. The functional assays we used in case of F3-LP, MBL-LP, CP, and AP detect the residual, inducible terminal complement complex generation in the given sample, as these tests are based on the *in vitro* activation of the samples using the specific activators of the F3-LP (acetylated bovine serum albumin), the MBL-LP (mannan), the CP (immune complexes) or the AP (lipopolysaccharide). Therefore, the observed higher *in vitro* activation capacity for F3-LP and AP activity found in the T2DM cohort indicate that these complement pathways have not been activated extensively *in vivo*. In accordance with the diminished *in vivo* activation in the T2DM patients, less increased sC5b-9 activation product concentrations were found in T2DM in comparison with the ND group. In addition, the observed higher C1-inhibitor concentrations along with the decreased C4d levels support a diminished *in vivo* F3-LP activation.

When we analyzed the F3-LP, AP, and CP pathways together, only 27% of diabetic patients had parallel activation in contrast to 48% of ND cases. This difference was mainly attributable to the diminished activation of F3-LP and AP in T2DM. However, one limitation of our study is that we do not have “baseline” (non-infectious) data regarding complement profile of the study subjects.

Regarding the different infection localizations, impaired F3-LP and AP activations were found in T2DM with urinary tract infections in comparison with the ND group. In diabetic patients having respiratory tract infections diminished activation of AP was seen, with a similar tendency regarding F3-LP activity. However, no changes were found in case of skin or soft tissue, or other types of infection locations between the two groups. Whether the observed difference in the altered activation of F3-LP and AP among the different localizations is explained by the different body compartments and/or the diversity of the etiological bacterial agents, still remains to be determined.

Recently, growing number of data have been published about the complement system and diabetes mellitus however, most of these studies have targeted chronic diabetes complications. Based on these observations activation of the complement system plays a role in diabetic vascular complications. C3 as a central component of complement system and its activation may contribute to chronic diabetic micro- and macrovascular complications (14–18). In a recent review, Ghosh and colleagues showed that increased complement activation may have an impact on the pathogenesis of diabetic vascular complications (19). Remarkably, higher sC5b-9 values and renal deposition in patients with diabetes were observed and were shown to be related with diabetic nephropathy (42). On the other hand, diminished complement-activating capacity through the classical pathway in type 2 diabetes mellitus was reported in the context of free sialic acid as a potential modulator of complement activation (20). It has to be noted, that in these studies subjects were involved in an infection-free period. Limited data were reported about the complement system during infections in hyperglycemic conditions: a previous study demonstrated that sera taken from healthy donors in hyperglycemic conditions *in vitro* (glucose concentration: 10–17 mmol/L) altered the interaction of C3 and the pathogenic bacteria *Staphylococcus aureus* (43). To the best of our knowledge our prospective study is the first publication about a complex investigation of complement activation during bacterial infections in diabetes compared to non-diabetic status. Based on our detailed measurements, diminished activation of F3-LP and AP in diabetic patients with bacterial infections was not due to lower levels of ficolins, C4 or C3, as antigenic concentrations of these complement components were similar in the two groups, or even higher in case of C3 in the T2DM group, in accordance with previous data (42, 44). Average concentrations of components of the lectin pathway (LP) were described previously (45–47) and several studies reported their levels during ongoing infections, but not in T2DM (48–50). Furthermore, it has to be highlighted, that concentrations of these components may vary age-dependently (47), and different comorbidities may have influence on them (51, 52), therefore, it remains a subject of research, how different pathogens or severity of infections could affect their levels.

High glucose related alterations in glycation pattern of complement components, or changes in tertiary structure of complement components may represent a potential explanation for the diminished F3-LP activation and AP amplification during infections of diabetic patients. C3 mediated effector functions were found to be inhibited in hyperglycemic conditions and the elegant study of Hair et al. showed that the inhibition is related to glucose induced changes in the tertiary structure of C3 (43). In our study blood glucose levels, short- (fructosamine), middle- (HbA_1c_) or long- (AGE) term glycation markers *per se* were elevated in the T2DM group compared to ND group. However, none of them were associated with activation and consumption of F3-LP and AP, except an inverse correlation of the F3-LP activity with the long-term glycation marker of AGEs in diabetic subjects. One possible explanation for the missing association with the short- and middle-term glycemic parameters could be that despite their higher levels in the T2DM group, our diabetic patients had a relatively good glycemic control around the time of the infection, as shown by the acceptable levels of blood glucose, fructosamine, and HbA_1c_.

We also evaluated associations of complement activation with clinical parameters and observed that 3-months post infection mortalities were similar in both groups. However, lack of ficolin-3-mediated lectin pathway activation along with lack of alternative pathway amplification were associated with mortality only in the T2DM group. There was no difference in the occurrence of complement consumption among the groups with sepsis without fatal outcome.

Throughout the various types of bacterial infections, the most remarkable difference between the two groups, regarding F3-LP and AP activation, was found in patients with UTI, especially in those suffering from *E. coli* infection. Ficolin-3 is one of the most effective activator of the LP *in vitro* (53) although our knowledge of ficolin-3 interaction with human pathogens is limited. Based on previous studies some human pathogens, such as *Hafnia alvei* (32) can activate the ficolin-3 mediated lectin pathway and ficolin-3 is linked with growth inhibition of *Aerococcus viridans* (54). Additionally, ficolin-3 recognized pathogenic *Pasteurella pneumotropica* and two pathogenic *E. coli* (enteroaggregative *E. coli* O71 and enteropathogenic *E. coli* O111 ab:H2) in an interaction study (31), but Sorensen et al. did not find any binding capacity of four prototypic enteroaggregative *E. coli* strains to recombinant ficolin-3 (55). Complement evasion strategies of *E. coli*, and altered factor H could also contribute to the diminished F3-LP activation and AP amplification in T2DM patients having *E. coli* related UTIs.

## Conclusions

In summary, we found impaired ficolin-3-mediated lectin pathway activation and decreased alternative pathway amplification during bacterial infections in patients with T2DM in comparison with non-diabetic individuals. Lack of ficolin-3-mediated lectin pathway and alternative pathway consumption in the T2DM group were associated with 3-months mortality. Less diabetic patients had parallel consumption of F3-LP, CP and AP compared to the non-diabetic subjects. Our data suggest that ficolin-3-mediated lectin and alternative pathway activations may have an impact on the clinical outcome of patients with type 2 diabetes having bacterial infections.

## Data Availability

The raw data supporting the conclusions of this manuscript will be made available by the authors, without undue reservation, to any qualified researcher.

## Ethics Statement

Study protocol was approved by the Hungarian Medical Research Council (TUKEB 396/2013 - 31584/2013/EKU) and the Institutional Review Board of the Semmelweis University, Budapest. Patients were involved into the study after informed, written consent in accordance with the Declaration of Helsinki.

## Author Contributions

ZP, ES, and NH designed and supervised the study. LB, ES, NH, and DC collected the blood samples. LB, KP, and DC performed ELISA, functional and hemolysis assays. LB, ES, and NH collected clinical and laboratory data. LB and ZP performed the statistical analysis. LB, ES, NH, ZP and DC wrote the manuscript with the help of KP and PG, who revised it critically for important intellectual content. All authors revised and approved the manuscript.

### Conflict of Interest Statement

The authors declare that the research was conducted in the absence of any commercial or financial relationships that could be construed as a potential conflict of interest. The reviewer AS and handling Editor declared their shared affiliation.
